# LncRNA H19 Overexpression Activates Wnt Signaling to Maintain the Hair Follicle Regeneration Potential of Dermal Papilla Cells

**DOI:** 10.3389/fgene.2020.00694

**Published:** 2020-08-04

**Authors:** Ningxia Zhu, En Lin, Huan Zhang, Yang Liu, Guiyuan Cao, Congcong Fu, Le Chen, Yang Zeng, Bozhi Cai, Yanping Yuan, Bin Xia, Keng Huang, Changmin Lin

**Affiliations:** ^1^Department of Pathophysiology, Guilin Medical University, Guilin, China; ^2^Department of Histology and Embryology, Shantou University Medical College, Shantou, China; ^3^Department of Reproductive Center, First Affiliated Hospital, Shantou University Medical College, Shantou, China; ^4^Tissue Engineering Laboratory, First Affiliated Hospital, Shantou University Medical College, Shantou, China; ^5^Department of Emergency, Second Affiliated Hospital, Shantou University Medical College, Shantou, China

**Keywords:** dermal papilla cell, H19, Wnt, alopecia, hair follicle

## Abstract

Androgenetic alopecia (AGA) is a common hair loss disorder resulting in seriously abnormal social interaction and psychological disorders. Transplantation with autologous dermal papilla cells represents a prospective therapy. However, the ability of dermal papilla cells to induce hair follicle development is lost upon cell culturing. Long non-coding RNAs (lncRNAs) are an important class of genes involved in various biological functions, are aberrantly expressed in disease and may play roles in the regulation of Wnt signaling, a critical pathway in maintaining the hair follicle-inducing capability of dermal papilla cells. Examination of dermal papilla cells by lncRNA microarray revealed that H19 was highly expressed in early passage dermal papilla cells compared with late-passage dermal papilla cells. In this study, we constructed H19-overexpressing dermal papilla cells to examine the role of H19 on hair follicle inductivity. Dermal papilla cells infected with lentivirus encoding H19 maintained their cell shape, and continued to display both multiple-layer aggregation and hair follicle-inducing ability upon prolonged culture. H19 exerted these effects through inducing miR-29a to activate Wnt signaling by directly downregulating the expression of Wnt suppressors, including DKK1, Kremen2, and sFRP2, thereby forming a novel regulatory feedback loop between H19 and miR-29a to maintain hair follicle- inducing potential. These results suggest that lncRNA H19 maintains the hair follicle-inducing ability of dermal papilla cells through activation of the Wnt pathway and could be a target for treatment of androgenetic alopecia.

## Introduction

Androgenetic alopecia (AGA) is an extremely common type of hair loss affecting the appearance and mental state in both men and women (Wang et al., 2010; Vincenzi et al., 2019). However, current treatments, including topical or systemic drugs and surgical transplantation, have numerous limitations due to severe side effects or availability of donor follicles. Cell-based hair regeneration offers a possible alternative, but current techniques are not capable of providing sufficient numbers of cells for regeneration (Ohn et al., 2019). Mesenchyme-derived dermal papilla (DP) cells, are located at the base of the hair follicle and regulate both development and growth of hair follicles, making them a potential therapeutic candidate for use in treatment of AGA (Ohyama et al., 2010; Ohn et al., 2019). However, although early passage DP cells can induce hair follicle regeneration *in vivo* and *vitro*, they quickly lose the hair inductivity during passaging *in vitro* (Jahoda et al., 1984; Yang and Cotsarelis, 2010).

Recent research demonstrates the importance of long non-coding RNAs (lncRNAs) in the regulation of various biological processes, such as cell development, differentiation, disease, subcellular localization and cellular structural maintenance (Wang and Chang, 2011; Kornienko et al., 2013; Boon et al., 2016). Also, for the biology of hair follicle, specific lncRNAs, such as ANCR, TINCR, HOTAIR, SPRY4-IT1 have been identified to be involved in the regulation of the HF cycle (Wan and Wang, 2014). Bao et al. (2017) reported a total of 2143 lncRNAs to be differentially expressed (fold change > 2.4) between AGA and adjacent normal tissues in a Chinese male population. Our previous study analyzed the differential expression pattern in passage-4 vs. passage-10 DP cells in a lncRNA microarray. Interestingly, results showed five transcripts (ENST00000442037, uc001va.4, uc021qbz1, ENST00000439725, and ENST00000417089) from H19 gene were highly expressed in early passage DP4 cells with hair follicle-inducing ability (Lin et al., 2014).

It is known that Wnt/β-catenin is a critical positive modulator in the maintenance of hair follicle regeneration by DP cells (Soma et al., 2012; Rishikaysh et al., 2014; Xiong et al., 2014) and H19 has been shown to play an important role in vascular smooth muscle cell proliferation and osteoblast differentiation by activating the Wnt/β-catenin pathway (Liang et al., 2016; Zhang et al., 2018). In addition, lncRNA H19 expression promotes bladder cancer metastasis by associating with EZH2 to activate the Wnt/β-catenin pathway (Luo et al., 2013). However, previous findings mainly focus on the mechanism of lncRNA in cancer and other diseases, or only on the expression patterns of lncRNAs in hair regeneration. Based on the high level expression of H19 in early passage cells and its close correlation with the Wnt signaling pathway, we used lentiviral transduction to overexpress H19 in DP cells and assessed viability and HF inducibility, as well as investigated the underlying regulatory mechanisms involving Wnt/β-catenin, to determine the role of H19 in DP cell-induced HF induction in this study.

## Materials and Methods

### Ethical Issues

The use of human scalp tissues was approved by the Ethics Committee of the First Affiliated Hospital, Shantou University Medical College. All participants provided written informed consent. Experiments with mice were performed with the permission of Shantou University Medical College.

### Isolation and Cultivation of DP Cells

Human scalp tissues were obtained from individuals undergoing forehead rhytidectomy or debridement and suturing. Primary culture and subculture of DP cells were performed as previously described (Li et al., 2005). DP cells were cultured in Dulbecco’s modified Eagle’s medium (DMEM) containing 10% fetal bovine serum (FBS, Invitrogen-Gibco) in a humidified atmosphere of 5% CO_2_ at 37°C, and were cultivated continuously. Cells used to confirm H19 expression and transfected with H19 overexpression plasmids were acquired from donors 8, 20, and 40 years of age, all of which were used at passage-4, passage-8, and passage-10. Cells for experiments applied to observe cell characteristics and infected with H19 overexpression lentivirus were obtained from donors 46, 50, 55 years of age, and all were used at passage 3–6 and 10.

#### Cell Transfection and Infection

The full-length H19 sequence was inserted into the pcDNA3.1 vector (pcDNA3.1-H19) (GenePharma, Shanghai, China). A plasmid carrying a non-targeting sequence (pcDNA3.1) was used as a negative control. DP cells were transfected using Lipofectamine 3000 reagent (Invitrogen, CA, United States). The protocols of plasmid construction and transfection are following the manufacturer’s instructions. Recombinant H19-expressing lentivirus encoding green fluorescent protein (GFP) (LV-H19) and the control lentiviral vector (GFP-lentivirus, LV-NC) were constructed by the GeneChem Company (Shanghai, China). To obtain stable cell lines, passage 3 DP cells were plated in 60 mm dishes and infected at a multiplicity of infection (MOI) of 20 when cell density reached 75% confluence. Medium was removed after 48 h and replaced with complete culture medium. Infection efficiency was confirmed by QPCR and fluorescence microscopic examination after infection. The primer sequences of pcDNA3.1-H19 and recombinant H19 lentiviral transfer vector were as follows: BamHI-F:CG CGGATCCAGTTAGAAAAAGCCCGGGCTAG; EcoRI-R:CC GGAATTCTTGCTGTAACAGTGTTTATTGATGATG. The detailed procedure of lentivirus construction is described in Supplementary File S1.

### Luciferase Reporter Assay

To evaluate the influence of H19 on the Wnt/β-catenin signaling pathway, a luciferase reporter plasmid pGL4.49[luc2P/TCF-LEF RE/Hygro] (Promega, United States) and pcDNA3.1/pcDNA3.1H19 as well as an internal control pGL4.74[hRluc/TK] vector (Promega, United States) were co-transfected into the passage 8 (DP8) cells at 70% confluence. After 24 h, luciferase reporter activity was measured with a Dual-Luciferase Assay System (Promega, Madison, WI, United States).

### RNA Extraction and Real-Time PCR

Total RNA, including lncRNA/miRNA fractions, was isolated from DP cells or dorsal skins, with a miRNeasy Mini Kit (QIAGEN, Beijing, China) and the quality of the RNA was assessed with a NanoDrop 2000 spectrophotometer (Thermo Fisher Scientific, United States) at 260 and 280 nm (A260/280). GAPDH was used as an internal control to normalize lncRNA/mRNA levels and U6 for MiR-29a-3p. Primers used for H19 and other mRNAs were provided by Invitrogen and the sequences are presented in Supplementary Tables S1, S2. Forward primers for miR-29a-3p and U6 were provided by TIANGEN Biotech (Beijing) Co., Ltd. RNA from the samples was reverse transcribed using a PrimeScript^TM^ RT reagent Kit with gDNA Eraser (Perfect Real Time) (TaKaRa, Dalian, China), or a miRcute miRNA First-Strand cDNA Synthesis kit (Tiangen, Beijing, China), according to the manufacturer’s instructions. qRT-PCR was performed using a standard SYBR^®^ Green PCR kit (RR820A, Toyobo Co. Ltd., Tokyo, Japan) or miRcute Plus miRNA qPCR Detection kit (Tiangen, Beijing, China) in a CFX96^TM^ Real-Time PCR Detection machine (Bio-Rad, United States). Changes in expression were determined by the 2^–ΔΔ^CT method.

### Animal Hair Follicle Induction

NU/NU mice (6 weeks old) were obtained from Beijing Vital River Laboratory Animal Technology Co., Ltd., and were divided into three groups: a control group injected with normal DP cells (Con-DP4), a control group injected with DP cells transfected with control lentivirus (LV-NC-DP4), and an experimental group injected with DP cells infected with H19-overexpressing lentivirus (LV-H19-DP4). About 3–5 × 10^4^ DP cells in 0.8 ml DMEM containing 10% fetal bovine serum were injected subcutaneously into each mouse dorsum. Mice were euthanized by CO_2_ inhalation after 10 days and the implantation sites were biopsied for molecular and histological analyses.

### Fluorescence *in situ* Hybridization

A Cy3-conjugated probe mix for lncRNA H19 was synthesized by GenePharma (Cy3-H19-Bio, Shanghai, China), and sequences were as follows (5′-3′): CTGTGCCTGCTACTAAATGA, ATGCTGTACTGTGCCAAG, ATGTCATGTCCTGTTGTCA, AAGCTAGAGGGTTTTGTGTC, CACACTCGTACTGAGAC TCA. Cells were fixed in 4% formaldehyde for 15 min, treated using 0.1% TritonX-100 for 15 min, and washed three times with PBS for 2 min, then incubated with 1× saline sodium citrate. Next, the cells were dehydrated using a graded ethanol series and incubated with the Cy3-labeled H19 probe mix at 37°C overnight, and washed with 1× saline sodium citrate four times for 3 min each wash. Lastly, nuclei were stained with DAPI. Signals were observed under a fluorescence microscope (Nikon H600L, Japan).

### Western Blot

For determining β-catenin levels in the nucleus and cytoplasm, nuclear, and cytoplasmic protein fractions of DP cells were extracted using a NE-PER Nuclear and Cytoplasmic Extraction Kit (78835; Pierce Biotechnology, United States). Total protein from DP cells, following H19 transient transfection, and dorsal skin tissues from nude mice were also extracted. The BCA method was applied for quantifying protein concentration. RIPA buffer was used to lyse cells and tissues and SDS-PAGE was conducted to separate the cellular or tissue proteins. Antibodies and dilutions used were as follows: GAPDH (Abcam, ab9485, United States, 1:5000), TBP (CST 8515S, United States, 1:1000), β-catenin (Abcam, ab32572, United States, 1:1000), DKK1 (Abcam, ab109416, United States, 1:1000), Kremen2 (Abcam, ab156007, United States, 1:1000), sFRP2 (Abcam, ab86379, United States, 1:1000), Wnt3a (1:1000, 09-162, Millipore), and LRP6 (1:1000, orb373345, Biorbyt). Bands were visualized with SuperSignal^TM^ West Femto Maximum Sensitivity Substrate (34095, Thermo Fisher Scientific) and a Universal Hood II gel imaging and image lab 3.0 analysis system (Bio-Rad, United States).

### Hematoxylin and Eosin (H&E) Staining

Dorsal skins from nude mice were harvested for histological analysis. Specimens were fixed with 4% paraformaldehyde, dehydrated through a graded series of ethanol, washed with xylene, and embedded in paraffin wax. Treated specimens were cut into 4 μm-thick sections and stained with hematoxylin and eosin for routine histology evaluation. Tissue was observed using bright field microscopy (Nikon H600L, Japan).

### Immunofluorescence Staining

Skin tissue sections were incubated with blocking solution containing 10% donkey serum for 30 min at 37°C and then probed overnight at 4°C with a diluted primary antibody, followed by a secondary antibody for 1 h. The primary antibodies used were: rabbit anti-β-catenin (1:200, ab32572, Abcam, Cambridge, United Kingdom), rabbit anti-Wnt3a (1:200, 09-162, Millipore), and rabbit anti-LRP6 (1:100, orb373345, Biorbyt). The secondary antibody used was Alexa Fluor 488-conjugated antibody (1:150, Jackson). DNA was stained with 4′, 6-diamidino-2-phenylindole (DAPI) (1 μg/ml, C1005, Beyotime, Jiangsu, China) for 10 min at room temperature. All images were collected using a fluorescence microscope (Nikon H600L, Japan).

### Biotin-Labeled RNA Pull-Down and Mass Spectrometry Analysis

RNA pull-down assay was performed as previously described (Bierhoff, 2018; Torres et al., 2018). Briefly, different segments of H19 were constructed with pcDNA3.1 plasmid, which also contains the T7 promoter. The plasmids were linearized using EcoRI. The anti-sense sequences are as follows: F:taatac gactcactatagggTTGCTGTAACAGTGTTTATTG; R:AGTTAG AAAAAGCCCGGGCTAG. RNA was precipitated and incubated with cell lysis buffer and biotin-labeled oligonucleotide for 2 h at RT. The RNA-protein complex was washed and eluted. The samples were separated using electrophoresis and identified by mass spectrometry, then sequences were retrieved in the Universal Protein Resource (UniProt) for human databases and the National Center for Biotechnology Information (NCBI) databases. To reduce the probability of false peptide identification, only peptides at a 95%, confidence interval (*P* < 0.05) with a false discovery rate (FDR) estimation 1.04% were counted as being successfully identified.

### Statistical Analysis

Data are expressed as mean ± SEM and analyzed by the two-tailed Student’s *t*-test or one-way analysis of variance (ANOVA). A *P* < 0.05 was considered statistically significant. All experiments were repeated at least three times, and for each experiment, samples were analyzed in triplicate.

## Results

### LncRNAH19 Differs Between Early and Late Passage DP Cells

**FIGURE 1:**
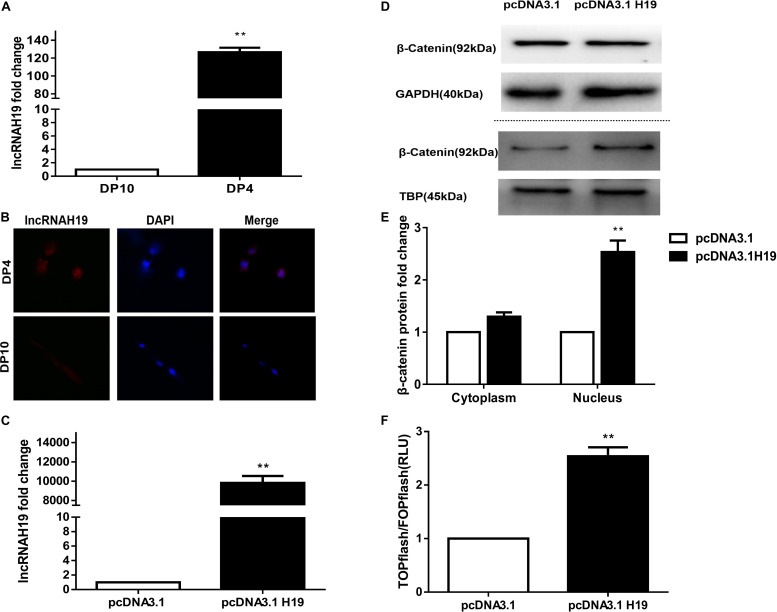
Differential expression of lncRNA H19 between early and late passage DP cells and effect of lncRNAH19 overexpression on the Wnt/β-catenin signaling pathway in DP cells. **(A)** QPCR verification the increased expression of lncRNAH19 in early passage DP cells (DP4) that show HF-inductivity ability. **(B)** FISH showing increased expression of lncRNAH19, in early passage DP cells (DP4), is mainly located in the nucleus (×40). **(C)** QPCR confirming that H19 was highly expressed in DP4 cells after being transfected with pcDNA3.1H19. **(D,E)** Compared with the pcDNA3.1 group, β-catenin expression in the nucleus was upregulated significantly after H19 overexpression (**B**, upper), whereas no obvious changes could be found in the expression of β-catenin in the cytoplasm (**B**, lower). **(F)** Luciferase reporter assay showed Wnt/β-catenin signaling-dependent reporter gene transcription was dramatically increased after H19 overexpression in DP8 cells. Data shown represent the mean ± SEM of three independent experiments. **P < 0.01, pcDNA3.1, mock-vehicle control group, pcDNA3.1H19 overexpression group.

In our previous lncRNA by microarray profiling (Lin et al., 2014) we reported that five transcripts from the H19 gene were up-regulated in early passage DP cells (DP4), which were HF-inducible. Here, we examined the lncRNA expression between early and late passage DP cells (DP4 and DP10) via QPCR and FISH. As shown in Figure 1, lncRNAH19 was increased in DP4 cells compared with that in DP10 cells (Figure 1A). Furthermore, high levels of lncRNAH19 were located in the nucleus of DP4 cells (Figure 1B). The above results suggested an involvement of H19 in regulating DP cell HF inducibility.

### Overexpression of LncRNAH19 Activates the Wnt/β-Catenin Signaling Pathway

The Wnt signaling pathway has been shown to be crucial for HF inducibility of DP cells and it is closely correlated with H19 (Soma et al., 2012; Lin et al., 2014; Rishikaysh et al., 2014; Xiong et al., 2014). Thus, we investigated the effect of H19 on the Wnt signaling pathway. Considering the slow proliferation rate and vulnerability of DP10 cells, we constructed an H19 expression plasmid (pcDNA3.1-H19), then transfected it or pcDNA3.1 empty vector into DP4 cells, then examined the expression and nuclear translocation of β-catenin and performed a Wnt reporter assay on DP8 cells. After 24 h, Results showed a significantly higher level of H19 gene expression in pcDNA3.1H19-transfected DP4 cells (Figure 1C). Furthermore, overexpression of H19 in DP4 cells upregulated β−catenin expression in the nucleus (Figures 1D,E). Additionally, TCF/LEF transcriptional activity of the luciferase reporter assay was increased by overexpressing H19 (Figure 1F). These results suggest lncRNA H19 activates the Wnt/β-catenin pathway to positively impact the DP cell HF inducibility.

### H19 Overexpression Activates Wnt Signaling by Upregulating MiR-29a-3p in DP Cells

**FIGURE 2:**
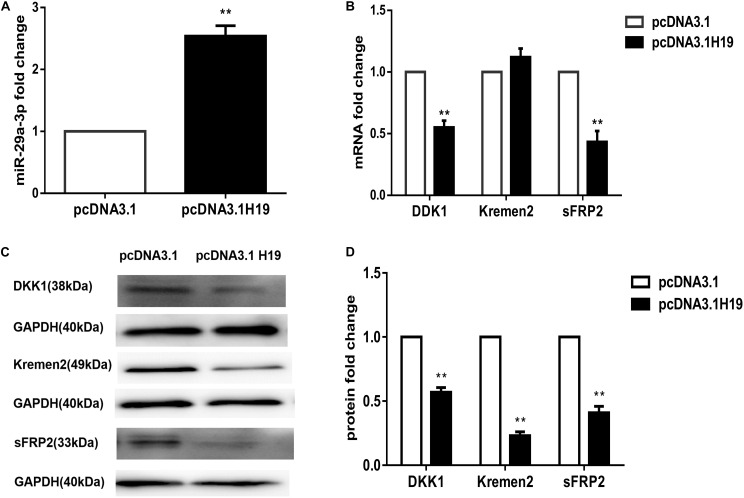
LncRNAH19 overexpression activates Wnt signaling by upregulating miR-29a-3p in DP4 cells *in vitro*. **(A)** LncRNAH19 overexpression upregulated the level of miR-29a-3p. **(B)** QPCR showed that DKK1 and sFRP2 were down-regulated, but Kremen2 mRNA expression was slightly increased when H19 was overexpressed in DP cells. **(C)** LncRNAH19 overexpression decreased Dkk1, Kremen2, and sFRP2 protein levels compared with the mock-vehicle group. **(D)** Quantification of Dkk1, Kremen2, and sFRP2 protein expression levels. Data shown represent the mean ± SEM of three independent experiments. **P < 0.01.

MiR-141, miR-22, and miR-29a were all reported to be target genes of H19 and are closely correlated to Wnt signaling (Jia et al., 2016; Gong et al., 2018; Wang et al., 2018). Our previous work showed miRNA-195-5p suppressed the Wnt/β-catenin pathway, possibly being responsible for the loss of HF-inducing ability of DP cells in culture (Zhu et al., 2018). We compared the expression of miR-141, miR-22, miRNA-195-5p, and miR-29a following transfection of the H19 plasmid into DP4 cells. Interestingly, miR-29a-3p expression was significantly elevated in H19-overexpressing DP cells (*P*< 0.01) (Figure 2A), while the others showed no change (data not shown). MiR-29a-3p target genes, such as Dikkopf-1 (DKK1), Kremen2, and secreted frizzled related protein 2 (sFRP2), are key factors contributing to inactivation of Wnt signaling (Kapinas et al., 2010; Nagano et al., 2013). We observed that mRNA levels of DKK1 and sFRP2 were significantly decreased, and Kremen2 was slightly increased in H19-overexpressing DP4 cells (Figure 2B). In addition, western blot analysis of Dkk1, Kremen2, and sFRP2 showed robust reductions in protein level compared with the mock-vehicle group (*P*< 0.01) (Figures 2C,D). The above results show that miR-29a-3p may be the H19 target through which H19 regulates Wnt signaling in DP cells.

### Overexpression of H19 Maintains DP Cell Viability *in vitro* and HF-Inducing Ability Upon Passaging

**FIGURE 3:**
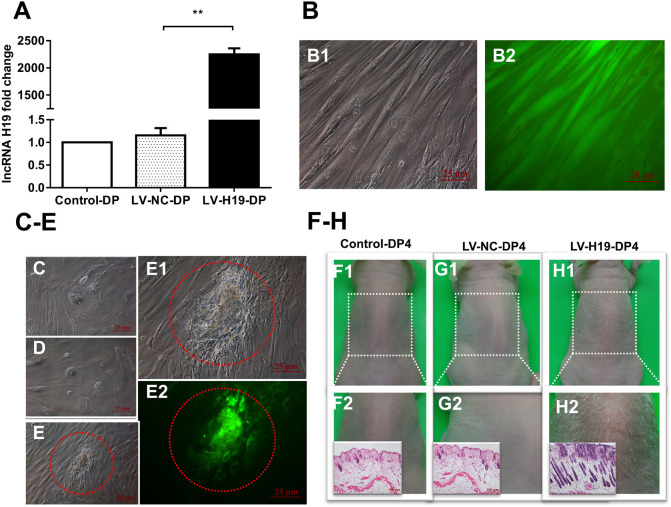
Effects of lncRNAH19 overexpression on maintenance of DP cell viability *in vitro* and HF-inducing ability *in vivo*. **(A)** H19 lentivirus upregulated the level of H19. **(B)** GFP fluorescent signal showed high infection efficiency (left: bright field; right: fluorescence, scale bar, 25 μm). **(C–E)** After infection of DP3 cells with LV-H19 and continuous culture, LV-H19-DP6 cells maintained aggravative behavior (e, red circle), but Control-DP6 or LV-NC-DP6 cells did not **(C,D)**. **(F–H)** LV-H19-DP4 cells induced hair generation on NU/NU mice backs after 10 days, with histological examination revealing HF structures, while the other two groups showed no hair growth. Data shown represent the mean ± SEM of three independent experiments. *n* = 8 mice per group. **P < 0.01. Control-DP4, normal DP4 cell-injected group, LV-NC-DP4, control virus-infected DP4 cell-injected group, LV-H19-DP4, the H19 overexpression lentivirus-infected DP4 cell-injected group.

To address the critical role of H19 in the HF-inducing ability of DP cells, we transduced DP cells, isolated from elderly patients, with H19 overexpression recombinant lentiviruses, then cultivated the cells continuously. Compared with DP cells used in above experiments, results showed that DP4 cells lost the aggregative behavior and HF-inducing ability when subcutaneously injected into the backs of mice, possibly due to the ages of the DP sources (data not shown). After infecting early passage DP cells (DP3) with recombinant lentiviruses, QPCR showed a significantly upregulated level of H19, and GFP fluorescence, indicating high infection efficiency (Figures 3A,B). Interestingly, the DP3 cells transduced with H19-expressing lentivirus still maintained a long fusiform, fibroblast-like shape at passage-6, which is crucial for their HF-inducing ability, whereas the control DP cells did not (Figures 3C–E).

Next, we tested for HF-inducing ability of DP4 cells infected with H19 lentiviruses at DP3. Mice were divided into three groups: a normal DP4 cell group (Control-DP4), a control virus-infected DP4 cell injection group (LV-NC-DP4), and an H19 lentivirus-infected DP4 cell injection group (LV-H19-DP4). After 10 days of treatment, a white hair coat with a hair length of 0.5–0.6 cm was observed in LV-H19-DP4-injected nude mice. Histology revealed large abnormal HF structures in the implanted sites, while control groups did not show any observable change (Figures 3F–H). Consequently, H19 expression inhibits the reductions in *in vitro* DP cell viability and *in vivo* HF-inducing ability that normally occur upon passage.

### LncRNA H19/MiR-29a Sustains HF-Inducing Effects of DP Cells *in vivo* by Regulating the Wnt Signaling Pathway

**FIGURE 4:**
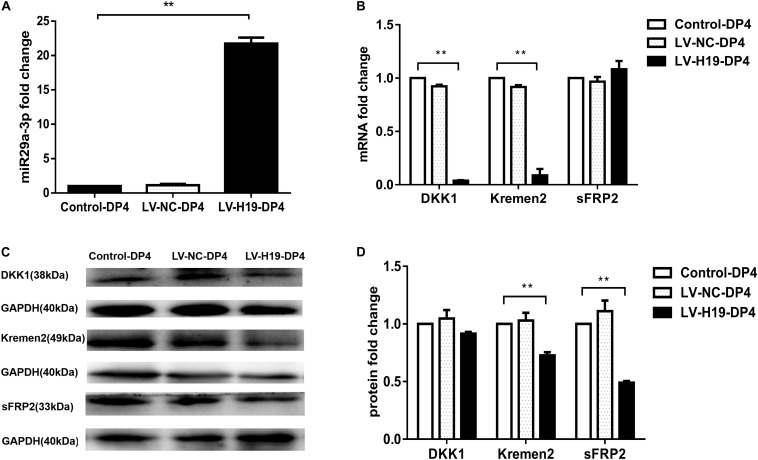
Overexpression of H19 upregulates miR-29a and reduces the expression of its target gene involvement in Wnt signaling in skin of NU/NU mice. **(A)** Injection of DP4 cells transfected with LV-H19 uprelated the level of miR-29a-3 in the skin. **(B)** QPCR showed that DKK1 and Kremen2 were down-regulated, but sFRP2 mRNA expression slightly increased following transduction of DP4 cells with LV-H19. **(C)** LncRNAH19 overexpression significantly decreased Kremen2, and sFRP2 protein levels, while the level of DKK1 was reduced slightly in the skin. **(D)** Dkk1, Kremen2, and sFRP2 protein expression levels were quantified. Data shown represent the mean ± SEM of three independent experiments. **P < 0.01.

**FIGURE 5:**
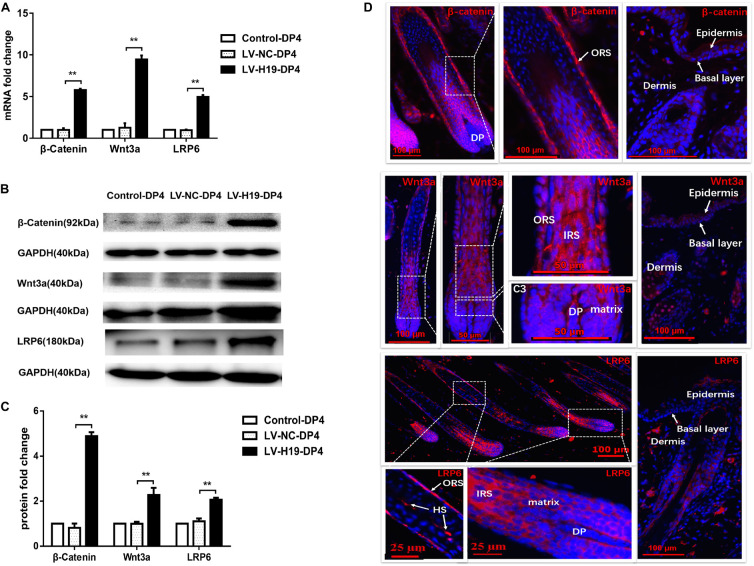
Expression pattern of Wnt3a, β-catenin and LRP6 in the back skin of NU/NU mice. **(A,B)** Both mRNA and protein levels of Wnt3a, β-catenin and LRP6 significantly increased when injected with LV-H19-infected DP4 cells. **(C)** Wnt3a, β-catenin and LRP6 protein expression levels were quantified. **(D)** Immunofluorescence staining showed Wnt3a, β-catenin and LRP6 were strongly expressed in the outer root sheath (ORS), inner root sheath (IRS), and matrix. No expression was found in the epithelial basal layer. HS, hair shaft. Data shown represent the mean ± SEM of three independent experiments. **P < 0.01.

To clarify whether miR-29a was involved in maintenance of HF inducibility *in vivo*, QPCR and western blot analysis were used to detect the RNA and protein expression patterns of miR-29a and its target genes after H19 overexpression by lentivirus transduction. Compared with control groups, miR-29a-3p expression was dramatically elevated (Figure 4A), and its target genes Dkk1 and Kremen2 were decreased at both the mRNA and protein levels in the LV-H19-DP4 group. Additionally, sFRP2 protein level was significantly reduced (Figures 4B–D). We also observed the expression pattern of β-catenin, Wnt3a and LRP6, key factors of Wnt/β-catenin signaling, in H19-expressing lentivirus-injected mouse dorsal skin. Results showed that β-catenin, Wnt3a and LRP6 mRNA and protein levels were significantly upregulated in the LV-H19-DP4 group (Figures 5A–C), whereas the location of β-catenin, Wnt3a, and LRP6 were mainly in the ORS, IRS and DP matrix, other than the epidermis, indicating an active anagen phase of the hair follicle cycle (Figure 5D). These results suggest an important role for H19/miR-29a/Wnt signaling in regulating DP cell-mediated HF induction.

### LncRNA H19 Combines With SAHH Protein in DP Cells

**FIGURE 6:**
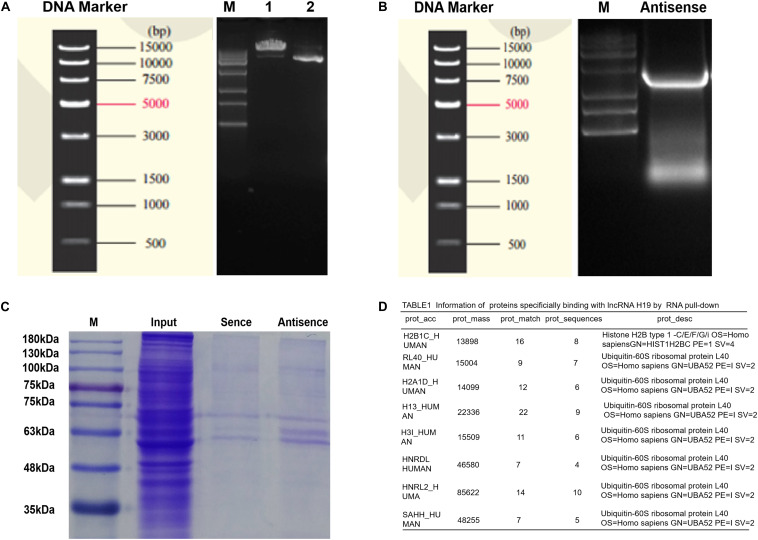
Determinations of proteins bound to lncRNA H19 by biotin-labeled RNA pull-down and mass spectrometry analysis in DP cells. **(A)** The results of plasmid linear enzyme digestion electrophoresis. **(B)** Electrophoretic results of H19 antisense PCR products. **(C)** Results of polyacrylamide gel electrophoresis (staining by Coomassie brilliant blue). **(D)** Information of proteins binding with lncRNA H19.

Research has shown H19 to bind and inhibit S-adenosylhomocysteine hydrolase (SAHH) to influence the methylation of targeted genes by DNMT, and thus may be a potential mechanism of regulating miR-29a (Zhou et al., 2015). To testify this, biotin-labeled RNA pull-down and mass spectrometry analysis were applied. First, electrophoresis confirmed the size of H19 pcDNA3.1 plasmid and H19 antisense chain PCR product, which were about 5428 and 2362 bp in length (Figures 6A,B). Secondly, Coomassie brilliant blue staining showed the differential protein bands between sense group and antisense group to be mainly located near 48 kDa (Figure 6C). To exclude non-specific binding, the results of mass spectrometry were searched in the database and compared with the results of the antisense chain. This resulted in 97 proteins showing specific binding with H19, including SAHH protein in human DP cells (Figure 6D and Supplementary Table S3).

## Discussion

Recently, DP cells have become an attractive means of treating of AGA in adult human HF regeneration, partly due to their easy expansion to meet urgent clinical requirements. Here, we show that lncRNA H19 maintains the competence of DP cells to induce HF growth by activating Wnt signaling, indicating H19 is a probable marker for hair loss in the early stages and could have potential for hair loss treatment. Jahoda et al. (1984) demonstrated that low-passage rodent DP cells exhibit HF-inducing capability, but gradually lose the ability during passaging. Also, they found intact human DP cells could induce HF regeneration, and Krugluger et al., 2005. proposed human DP cells at low passage could induce HF-like structure formation in human skin when co-cultured with outer sheath keratinocytes (Jahoda et al., 2001; Krugluger et al., 2005). Interestingly, histopathologic examination has found nude mice injected with adult human DP cells show evidence of hair growth, but fail to show hair emerging from the injected site (Nilforoushzadeh et al., 2017). Our study demonstrates early passage human DP cells (DP1-4) show HF-inducing capability 10 days after injection into the back of NU/NU mice (Lin et al., 2014). The probable explanations for the observed differences between the reported results are the donor’s age and species used. Also, in this study, H19-overexpressiong DP4 cells from elderly donors, showed no HF inductive ability, whereas in our study and H19 overexpression experiments, the DP4 cells were HF-inducing. Differences could possibly involve the mechanism of senescence. It has been reported that DP cells from balding scalps undergo pre-senescence, and show higher senescence-associated β-Gal activity (Upton et al., 2015) whereas inhibiting p21 activated kinase 1 (PAK1) indicated growth-promoting activity (Nozomi et al., 2017).

In a previous study, we determined a high expression level of lncRNA H19 together with HOTAIR by microarray analysis in DP cells (Lin et al., 2014) and the present study confirms their expression pattern by QPCR. We found that H19 is significantly up-regulated in early passage DP cells, which is consistent with the microarray results, but HOTAIR is unchanged (data not shown). Moreover, FISH showed increased levels of lncRNAH19, identical to the microarray results, which is mainly located in the nucleus in DP4 cells. Furthermore, we investigated the effect of H19 expression on DP cell viability *in vitro* and HF-inducing ability concomitant with cell passage *in vivo*. In the experimental group, after H19 was overexpressed from 3rd passage DP cells onward, the cell shape, proliferation and multi-layer aggregation were maintained until passage-6. Notably, the ability of DP cells to induce HF formation is dependent on their aggregative growth (Cheng et al., 2017). Moreover, HF-inducing ability is observed with 4th passage DP cells, overexpressing H19, but not with control DP cells in passage 4, following injection into NU/NU mice. Using high-yield extracellular vesicle-mimetic nano-vesicles (EMNVs) as an effective nano-drug delivery system for lncRNA, EMNVs with a high content of lncRNA-H19 (H19EMNVs) induced structures resembling HFs (Tao et al., 2018). LncRNA-H19 transcript expression is dramatically higher at the anagen phase than that at both telogen and catagen phase in goats (Zhu et al., 2017). However, knockdown of H19 with siRNA was difficult to observe reduced expression pattern, possibly due to siRNA knockdown of lncRNAs can be difficult according to location or secondary structure in specific DP cells (data not shown). Nevertheless, our results indicate the potential of H19 in maintaining the HF-inducing competence of DP cells.

The Wnt/β-catenin signaling pathway plays an important role in promoting HF regeneration in DP cells (Soma et al., 2012; Rishikaysh et al., 2014; Xiong et al., 2014). Emerging evidence suggests a close correlation between H19 and the Wnt/β-catenin pathway (Liang et al., 2016; Li et al., 2017; Zhang et al., 2017, 2018). Our results suggest β-catenin is increased in the nucleus and TCF/LEF transcriptional activity is significantly elevated, indicative of an activated Wnt signaling pathway in H19-overexpressing DP cells (Komiya and Habas, 2008). Additionally, β-catenin, Wnt3a and LRP6 levels are upregulated in HFs formed from LV-H19-DP4 cells, and the locations are mainly in the ORS, IRS and DP matrix, other than the epidermis, indicating activation of anagen phase of the hair follicle by H19 (Lin et al., 2015; Rognoni et al., 2016).

**FIGURE 7:**
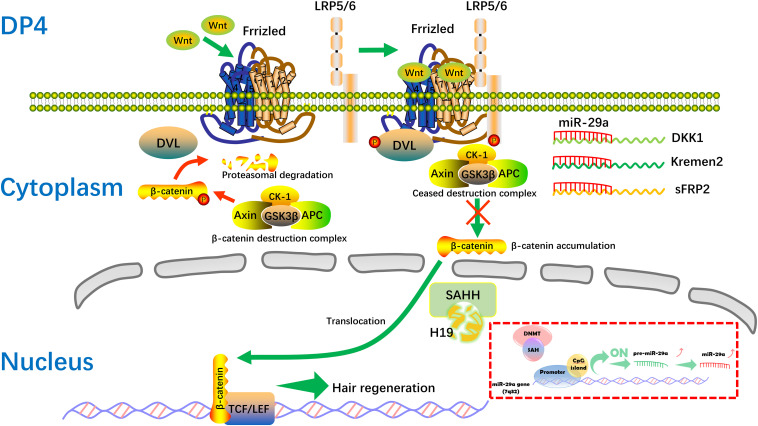
Hypothetical molecular mechanism of the H19/miR-29a/Wnt signaling pathway in HF inducibility of DP cells. When H19 is overexpressed, SAHH activity is attenuated, leading to accumulation of SAH, which binds to DNMT and prevents it from methylating DNA. MiR-29a directly suppresses the expression of Dkk1, Kremen2, and sFRP2, which act as suppressors of the Wnt pathway, thereby forming a novel regulatory feedback loop between H19 and miR-29a to mediate HF regeneration. SAH, S-adenosylhomocysteine; SAHH, S-adenosylhomocysteine hydrolase.

The mechanisms mediated by lncRNAs include chromatin modification, genomic imprinting, and functioning as sponge regulators of miRNA to target genes (Akhade et al., 2017). Here, we found the level of miR-29a is increased after H19 overexpression in DP cells. In addition, high levels of miR-29a directly suppress the expression of Dkk1, Kremen2, and sFRP2, which are suppressors of the Wnt pathway, thus activating the Wnt signaling pathway, in accordance with previous studies (Kapinas et al., 2010; Nagano et al., 2013). Still, miR-29a has been reported to inhibit hair follicle stem cell lineage progression by spatiotemporally suppressing Wnt and BMP Signaling (Ge et al., 2019). The discrepancy is probably due to the different cells, different animal models, or intervention strategy used in the research. Also, there maybe exist other regulatory mechanisms between H19 and miR-29a. Compared with the inhibiting effect of lncRNAH19 on miRNAs (Jia et al., 2016; Lu et al., 2016; He et al., 2017). H19 associates with the hnRNP U/PCAF/RNA Pol II protein complex to positively regulate the miR-200 family by increasing histone acetylation (Zhang et al., 2013). SAHH is shown to be the only enzyme to hydrolyze S-adenosylhomocysteine (SAH) in mammals, which acts as an inhibitor of methyl reaction (Zhou et al., 2015). Interestingly, H19 has been indicated to bind and inhibit SAHH, and H19 knockdown activates SAHH, leading to increased DNMT-mediated methylation of a targeted gene. Genome-wide methylation profiling has revealed that methylation changes at numerous gene loci, consistent with SAHH modulation by H19 (Zhou et al., 2015). DNA methylation is generally associated with chromatin condensation, which plays an important role in silencing genes (Ikegami et al., 2009). Also, epigenetic gene inactivation associated with methylation of promoter CpG-islands is common to miRNA genes (Loginov et al., 2015). Studies show expression of miRNA genes is also regulated via epigenetic mechanisms, including CpG-island methylation (Kozaki et al., 2008; Ando et al., 2009). According to a number of publications, methylation is involved in regulating more than 11% of miRNA genes (Kunej et al., 2011; Loginov et al., 2015). Of relevance, the miR-29 family has been found to regulate DNA methylation pathways (Fabbri et al., 2007; Garzon et al., 2009; Robaina et al., 2015). In our study, RNA pull-down results showed that there is specific binding between H19 and SAHH in DP cells, which is identical to a previous study (Zhou et al., 2015). Taken together, we hypothesize that when H19 is overexpressed, SAHH activity is attenuated, which leads to accumulation of SAH and preventing DNMT from methylating the CpG island within the miR-29a gene promoter (Jiang et al., 2014). The miR-29a gene, being methylated in non-HF-inducing DP cells, becomes demethylated and is actively expressed. Restored expression of miR-29a in non-HF-inducing DP cells would result in enhanced potential to maintain hair follicle regeneration, particularly through reducing expression of the DKK1, sFRP2, and KREMEN2, which are inhibitors of the Wnt/ß-catenin signaling pathway. Further studies will be engaged to describe the mechanism of regulation between H19 and miR-29a in DP cells (Figure 7).

## Conclusion

In conclusion, this study explores the crucial role of H19 and miR-29a in the Wnt signaling pathway pertaining to HF regeneration *in vitro* and *vivo*, suggesting a possible strategy for improving the therapy of AGA patients. Thus, more comprehensive functional and mechanistic studies will be necessary and worthwhile.

## Data Availability Statement

All datasets generated for this study are included in the article/Supplementary Material.

## Ethics Statement

The studies involving human participants were reviewed and approved by the Ethics Committee of the First Affiliated Hospital. Written informed consent to participate in this study was provided by the participants’ legal guardian/next of kin. The animal study was reviewed and approved by Ethics Committee of the Shantou University Medical College.

## Author Contributions

NZ, EL, and HZ performed the experiments. YL, GC, CF, and LC helped to analyze and interpret the data. YZ, BC, YY, and BX helped to analyze the data and draft the manuscript. KH and CL conceived and designed the study and drafted the manuscript. All authors contributed to the article and approved the submitted version.

## Conflict of Interest

The authors declare that the research was conducted in the absence of any commercial or financial relationships that could be construed as a potential conflict of interest.

## Supplementary Material

TABLE S1 Primers used to detect the expression of lncRNA H19/mRNAs in human DP cells.

TABLE S2 Primers used to detect the expression of mRNAs in dorsal skin in NU/NU mice.

TABLE S3 Proteins binding with lncRNA H19 by biotin-labeled RNA pull-down and mass spectrometry analysis in DP cells (the figures in the Match an Sequence columns represent the number of scores higher than Confidence Threshold).

FILE S1 The detailed procedure of H19 overexpression lentivirus construction.

## Abbreviations

DP: dermal papilla
HF: hair follicle
lncRNA: long non-coding RNA
miRNA/miR: microRNA
WB: western blot
FISH: fluorescence *in situ* hybridization
LV: lentivirus
NC: negative control
DKK1: Dickkopf-1
Kremen2: Kringle containing transmembrane protein 2
sFRP2: secreted frizzled-related protein 2
SAHH: S-adenosylhomocysteine hydrolase
SEM: standard error of mean.
