# Supplementary material for: LncRNA H19 Overexpression Activates Wnt Signaling to Maintain the Hair Follicle Regeneration Potential of Dermal Papilla Cells
Source: Front Genet. 2020 Aug 4;11:694. doi: 10.3389/fgene.2020.00694 (PMC7417632; doi:10.3389/fgene.2020.00694)
Supplement: TABLE S1 — Primers used to detect the expression of lncRNA H19/mRNAs in human DP cells. [file Table_1.DOC]

TABLE 1 Primers used to detect the expression of lncRNA H19/mRNAs in human DP cells.

| H19 | F：5’CCAGCCACCACATCATCC 3’  R：5’GACTCAAGGCCGTCTCCAC 3’ |
| --- | --- |
| WIF1 | F：5’AGTGTCCTGATGGGTTCCAC 3’  R：5’TGGTTGAGCAGTTTGCTTTG 3’ |
| DKK1 | F：5’TCCGAGGAGAAATTGAGGAA 3’  R：5’CCTGAGGCACAGTCTGATGA 3’ |
| Kremen2 | F：5’CGGGTAGGGACAGGGACAGA 3’  R：5’GCGGGAGGAAGAGGAGAAAGA 3’ |
| sFRP2 | F：5’GCCTCGATGACCTAGACGAG 3’  R：5’GATGCAAAGGTCGTTGTCC 3’ |
| GAPDH | F：5’ACCTGACCTGCCGTCTAGAA 3’  R：5’TCCACCACCCTGTTGCTGTA 3’ |
