# Supplementary material for: LncRNA H19 Overexpression Activates Wnt Signaling to Maintain the Hair Follicle Regeneration Potential of Dermal Papilla Cells
Source: Front Genet. 2020 Aug 4;11:694. doi: 10.3389/fgene.2020.00694 (PMC7417632; doi:10.3389/fgene.2020.00694)
Supplement: TABLE S2 — Primers used to detect the expression of mRNAs in dorsal skin in NU/NU mice. [file Table_2.DOC]

**TABLE 2** Primers used to detect the expression of mRNAs in dorsal skin in NU/NU mice.

| GAPDH | F：5’GTGAAGGTCGGTGTGAACGGATT3’ |
| --- | --- |
|  | R：5’GGTCTCGCTCCTGGAAGATGGT3’ |
| β-catenin | F：5’GACAAGCCACAGGATTACAAG3’ |
|  | R：5’CCAGAGTGAAAAGAACGGTAG3’ |
| Wnt3a | F：5’ATGGCTCCTCTCGGATACCT3’ |
|  | R：5’GGGCATGATCTCCACGTAGT3’ |
| LRP6 | F：5’TGCAAACTCAGTCGCAAATC3’ |
|  | R：5’CTTTCTCGGGGTTTACCACA3’ |
| LEF-1 | F：5’ACCTACAGCGACGAGCACT3’ |
|  | R：5’CGTGTTGAGGCTTCACGTGC3’ |
| TCF3 | F：5’GGGCTCTGACAAGGAACTGA3’ |
|  | R：5’CAAAGGTGGCATAGGCATT3’ |
| DKK1 | F：5’CTGAAGATGAGGAGTGCGGCTC3’ |
|  | R：5’GGCTGTGGTCAGAGGGCATG3’ |
| sFRP2 | F：5’TCATGCAATGAGGAATGGTT3’ |
|  | R：5’TTTCGTTGCTCGTTTTATGC3’ |
| Kremen2 | F：5’AGGGAAACTGGTCGGCTC3’ |
|  | R：5’AAGGCACGGAGTAGGTTGC3’ |
