# Supplementary material for: LncRNA H19 Overexpression Activates Wnt Signaling to Maintain the Hair Follicle Regeneration Potential of Dermal Papilla Cells
Source: Front Genet. 2020 Aug 4;11:694. doi: 10.3389/fgene.2020.00694 (PMC7417632; doi:10.3389/fgene.2020.00694)
Supplement: TABLE S3 — Proteins binding with lncRNA H19 by biotin-labeled RNA pull-down and mass spectrometry analysis in DP cells (the figures in the Match an Sequence columns represent the number of scores higher than Confidence Threshold). [file Table_3.doc]

**TABLE 3** Proteins binding to lncRNA H19, determined by biotin-labeled RNA pull-down and

mass spectrometry analysis in DP cells.

| **prot_number** | **prot_access number** | **prot_mass** | **prot_score** | **prot_matches** | **prot_sequences** | **prot_abundance** | **prot_descriptions** |
| --- | --- | --- | --- | --- | --- | --- | --- |
| 47.1 | [H2B1C_HUMAN](http://fitgene-cell-pc/mascot/cgi/master_results_2.pl?file=20180201/F022332.dat;pr.eh=47,47p;pr.page=47;pr.per_page=1;pr.show=proteins) | 179 | 13898 | 16 (11) | 8 (6) | 4.8 | Histone H2B type 1-C/E/F/G/I OS=Homo sapiens GN=HIST1H2BC PE=1 SV=4 |
| 62 | [RL40_HUMAN](http://fitgene-cell-pc/mascot/cgi/master_results_2.pl?file=20180201/F022332.dat;pr.eh=62,62p;pr.page=62;pr.per_page=1;pr.show=proteins) | 142 | 15004 | 9 (6) | 7 (4) | 1.27 | Ubiquitin-60S ribosomal protein L40 OS=Homo sapiens GN=UBA52 PE=1 SV=2 |
| 117 | [H2A1D_HUMAN](http://fitgene-cell-pc/mascot/cgi/master_results_2.pl?file=20180201/F022332.dat;pr.eh=117,117p;pr.page=117;pr.per_page=1;pr.show=proteins) | 67 | 14099 | 12 (7) | 6 (3) | 0.91 | Histone H2A type 1-D OS=Homo sapiens GN=HIST1H2AD PE=1 SV=2 |
| 28.2 | [TBA1A_HUMAN](http://fitgene-cell-pc/mascot/cgi/master_results_2.pl?file=20180201/F022332.dat;pr.eh=28,28p;pr.page=28;pr.per_page=1;pr.show=proteins) | 273 | 50788 | 25 (13) | 10 (9) | 0.76 | Tubulin alpha-1A chain OS=Homo sapiens GN=TUBA1A PE=1 SV=1 |
| 114.1 | [H13_HUMAN](http://fitgene-cell-pc/mascot/cgi/master_results_2.pl?file=20180201/F022332.dat;pr.eh=114,114p;pr.page=114;pr.per_page=1;pr.show=proteins) | 68 | 22336 | 22 (3) | 9 (3) | 0.52 | Histone H1.3 OS=Homo sapiens GN=HIST1H1D PE=1 SV=2 |
| 145 | [H31_HUMAN](http://fitgene-cell-pc/mascot/cgi/master_results_2.pl?file=20180201/F022332.dat;pr.eh=145,145p;pr.page=145;pr.per_page=1;pr.show=proteins) | 49 | 15509 | 11 (3) | 6 (2) | 0.48 | Histone H3.1 OS=Homo sapiens GN=HIST1H3A PE=1 SV=2 |
| 135 | [RSMN_HUMAN](http://fitgene-cell-pc/mascot/cgi/master_results_2.pl?file=20180201/F022332.dat;pr.eh=135,135p;pr.page=135;pr.per_page=1;pr.show=proteins) | 52 | 24769 | 5 (3) | 5 (3) | 0.46 | Small nuclear ribonucleoprotein-associated protein N OS=Homo sapiens GN=SNRPN PE=1 SV=1 |
| 204 | [LSM3_HUMAN](http://fitgene-cell-pc/mascot/cgi/master_results_2.pl?file=20180201/F022332.dat;pr.eh=204,204p;pr.page=204;pr.per_page=1;pr.show=proteins) | 35 | 11838 | 1 (1) | 1 (1) | 0.29 | U6 snRNA-associated Sm-like protein LSm3 OS=Homo sapiens GN=LSM3 PE=1 SV=2 |
| 126 | [1433F_HUMAN](http://fitgene-cell-pc/mascot/cgi/master_results_2.pl?file=20180201/F022332.dat;pr.eh=126,126p;pr.page=126;pr.per_page=1;pr.show=proteins) | 59 | 28372 | 7 (2) | 5 (2) | 0.25 | 14-3-3 protein eta OS=Homo sapiens GN=YWHAH PE=1 SV=4 |
| 36.2 | [HNRDL_HUMAN](http://fitgene-cell-pc/mascot/cgi/master_results_2.pl?file=20180201/F022332.dat;pr.eh=36,36p;pr.page=36;pr.per_page=1;pr.show=proteins) | 106 | 46580 | 7 (5) | 4 (3) | 0.23 | Heterogeneous nuclear ribonucleoprotein D-like OS=Homo sapiens GN=HNRNPDL PE=1 SV=3 |
| 302 | [FABP9_HUMAN](http://fitgene-cell-pc/mascot/cgi/master_results_2.pl?file=20180201/F022332.dat;pr.eh=302,302p;pr.page=302;pr.per_page=1;pr.show=proteins) | 21 | 15140 | 5 (1) | 4 (1) | 0.23 | Fatty acid-binding protein 9 OS=Homo sapiens GN=FABP9 PE=1 SV=1 |
| 353 | [RU1C_HUMAN](http://fitgene-cell-pc/mascot/cgi/master_results_2.pl?file=20180201/F022332.dat;pr.eh=353,353p;pr.page=353;pr.per_page=1;pr.show=proteins) | 15 | 17552 | 1 (1) | 1 (1) | 0.19 | U1 small nuclear ribonucleoprotein C OS=Homo sapiens GN=SNRPC PE=1 SV=1 |
| 207 | [CU086_HUMAN](http://fitgene-cell-pc/mascot/cgi/master_results_2.pl?file=20180201/F022332.dat;pr.eh=207,207p;pr.page=207;pr.per_page=1;pr.show=proteins) | 35 | 18909 | 2 (1) | 2 (1) | 0.18 | Putative uncharacterized protein encoded by LINC00205 OS=Homo sapiens GN=LINC00205 PE=5 SV=1 |
| 239 | [1B47_HUMAN](http://fitgene-cell-pc/mascot/cgi/master_results_2.pl?file=20180201/F022332.dat;pr.eh=239,239p;pr.page=239;pr.per_page=1;pr.show=proteins) | 29 | 40888 | 4 (2) | 4 (2) | 0.17 | HLA class I histocompatibility antigen, B-47 alpha chain OS=Homo sapiens GN=HLA-B PE=1 SV=1 |
| 128 | [HNRL2_HUMAN](http://fitgene-cell-pc/mascot/cgi/master_results_2.pl?file=20180201/F022332.dat;pr.eh=128,128p;pr.page=128;pr.per_page=1;pr.show=proteins) | 58 | 85622 | 14 (4) | 10 (4) | 0.16 | Heterogeneous nuclear ribonucleoprotein U-like protein 2 OS=Homo sapiens GN=HNRNPUL2 PE=1 SV=1 |
| 276 | [ARF3_HUMAN](http://fitgene-cell-pc/mascot/cgi/master_results_2.pl?file=20180201/F022332.dat;pr.eh=276,276p;pr.page=276;pr.per_page=1;pr.show=proteins) | 25 | 20645 | 4 (1) | 4 (1) | 0.16 | ADP-ribosylation factor 3 OS=Homo sapiens GN=ARF3 PE=1 SV=2 |
| 156 | [RAB1C_HUMAN](http://fitgene-cell-pc/mascot/cgi/master_results_2.pl?file=20180201/F022332.dat;pr.eh=156,156p;pr.page=156;pr.per_page=1;pr.show=proteins) | 45 | 22231 | 6 (2) | 5 (1) | 0.15 | Putative Ras-related protein Rab-1C OS=Homo sapiens GN=RAB1C PE=5 SV=2 |
| 319 | [PGRC1_HUMAN](http://fitgene-cell-pc/mascot/cgi/master_results_2.pl?file=20180201/F022332.dat;pr.eh=319,319p;pr.page=319;pr.per_page=1;pr.show=proteins) | 20 | 21772 | 1 (1) | 1 (1) | 0.15 | Membrane-associated progesterone receptor component 1 OS=Homo sapiens GN=PGRMC1 PE=1 SV=3 |
| 240 | [RU2B_HUMAN](http://fitgene-cell-pc/mascot/cgi/master_results_2.pl?file=20180201/F022332.dat;pr.eh=240,240p;pr.page=240;pr.per_page=1;pr.show=proteins) | 28 | 25470 | 4 (1) | 4 (1) | 0.13 | U2 small nuclear ribonucleoprotein B'' OS=Homo sapiens GN=SNRPB2 PE=1 SV=1 |
| 259 | [RU17_HUMAN](http://fitgene-cell-pc/mascot/cgi/master_results_2.pl?file=20180201/F022332.dat;pr.eh=259,259p;pr.page=259;pr.per_page=1;pr.show=proteins) | 26 | 51583 | 4 (2) | 3 (2) | 0.13 | U1 small nuclear ribonucleoprotein 70 kDa OS=Homo sapiens GN=SNRNP70 PE=1 SV=2 |
| 352 | [PSMD9_HUMAN](http://fitgene-cell-pc/mascot/cgi/master_results_2.pl?file=20180201/F022332.dat;pr.eh=352,352p;pr.page=352;pr.per_page=1;pr.show=proteins) | 15 | 24838 | 2 (1) | 2 (1) | 0.13 | 26S proteasome non-ATPase regulatory subunit 9 OS=Homo sapiens GN=PSMD9 PE=1 SV=3 |
| 220 | [PCLI1_HUMAN](http://fitgene-cell-pc/mascot/cgi/master_results_2.pl?file=20180201/F022332.dat;pr.eh=220,220p;pr.page=220;pr.per_page=1;pr.show=proteins) | 32 | 28766 | 3 (1) | 2 (1) | 0.12 | PTB-containing, cubilin and LRP1-interacting protein OS=Homo sapiens GN=PID1 PE=1 SV=1 |
| 254 | [GDF5O_HUMAN](http://fitgene-cell-pc/mascot/cgi/master_results_2.pl?file=20180201/F022332.dat;pr.eh=254,254p;pr.page=254;pr.per_page=1;pr.show=proteins) | 27 | 28422 | 5 (1) | 2 (1) | 0.12 | Protein GDF5OS, mitochondrial OS=Homo sapiens GN=GDF5OS PE=2 SV=2 |
| 223 | [DECR2_HUMAN](http://fitgene-cell-pc/mascot/cgi/master_results_2.pl?file=20180201/F022332.dat;pr.eh=223,223p;pr.page=223;pr.per_page=1;pr.show=proteins) | 31 | 31100 | 6 (1) | 6 (1) | 0.11 | Peroxisomal 2,4-dienoyl-CoA reductase OS=Homo sapiens GN=DECR2 PE=1 SV=1 |
| 270 | [CH60_HUMAN](http://fitgene-cell-pc/mascot/cgi/master_results_2.pl?file=20180201/F022332.dat;pr.eh=270,270p;pr.page=270;pr.per_page=1;pr.show=proteins) | 26 | 61187 | 8 (2) | 7 (2) | 0.11 | 60 kDa heat shock protein, mitochondrial OS=Homo sapiens GN=HSPD1 PE=1 SV=2 |
| 327 | [OTUB1_HUMAN](http://fitgene-cell-pc/mascot/cgi/master_results_2.pl?file=20180201/F022332.dat;pr.eh=327,327p;pr.page=327;pr.per_page=1;pr.show=proteins) | 18 | 31492 | 4 (1) | 2 (1) | 0.11 | Ubiquitin thioesterase OTUB1 OS=Homo sapiens GN=OTUB1 PE=1 SV=2 |
| 336 | [MTX2_HUMAN](http://fitgene-cell-pc/mascot/cgi/master_results_2.pl?file=20180201/F022332.dat;pr.eh=336,336p;pr.page=336;pr.per_page=1;pr.show=proteins) | 17 | 30086 | 2 (1) | 1 (1) | 0.11 | Metaxin-2 OS=Homo sapiens GN=MTX2 PE=1 SV=1 |
| 177 | [FXR1_HUMAN](http://fitgene-cell-pc/mascot/cgi/master_results_2.pl?file=20180201/F022332.dat;pr.eh=177,177p;pr.page=177;pr.per_page=1;pr.show=proteins) | 39 | 70020 | 5 (2) | 5 (2) | 0.1 | Fragile X mental retardation syndrome-related protein 1 OS=Homo sapiens GN=FXR1 PE=1 SV=3 |
| 274 | [DHI1L_HUMAN](http://fitgene-cell-pc/mascot/cgi/master_results_2.pl?file=20180201/F022332.dat;pr.eh=274,274p;pr.page=274;pr.per_page=1;pr.show=proteins) | 25 | 34438 | 2 (1) | 2 (1) | 0.1 | Hydroxysteroid 11-beta-dehydrogenase 1-like protein OS=Homo sapiens GN=HSD11B1L PE=2 SV=1 |
| 337 | [PACRG_HUMAN](http://fitgene-cell-pc/mascot/cgi/master_results_2.pl?file=20180201/F022332.dat;pr.eh=337,337p;pr.page=337;pr.per_page=1;pr.show=proteins) | 17 | 33662 | 1 (1) | 1 (1) | 0.1 | Parkin coregulated gene protein OS=Homo sapiens GN=PACRG PE=1 SV=2 |
| 150 | [ADT4_HUMAN](http://fitgene-cell-pc/mascot/cgi/master_results_2.pl?file=20180201/F022332.dat;pr.eh=150,150p;pr.page=150;pr.per_page=1;pr.show=proteins) | 47 | 35285 | 8 (1) | 6 (1) | 0.09 | ADP/ATP translocase 4 OS=Homo sapiens GN=SLC25A31 PE=2 SV=1 |
| 250 | [DHB3_HUMAN](http://fitgene-cell-pc/mascot/cgi/master_results_2.pl?file=20180201/F022332.dat;pr.eh=250,250p;pr.page=250;pr.per_page=1;pr.show=proteins) | 27 | 34950 | 1 (1) | 1 (1) | 0.09 | Testosterone 17-beta-dehydrogenase 3 OS=Homo sapiens GN=HSD17B3 PE=1 SV=2 |
| 260 | [ECH1_HUMAN](http://fitgene-cell-pc/mascot/cgi/master_results_2.pl?file=20180201/F022332.dat;pr.eh=260,260p;pr.page=260;pr.per_page=1;pr.show=proteins) | 26 | 36136 | 3 (1) | 3 (1) | 0.09 | Delta(3,5)-Delta(2,4)-dienoyl-CoA isomerase, mitochondrial OS=Homo sapiens GN=ECH1 PE=1 SV=2 |
| 322 | [S2533_HUMAN](http://fitgene-cell-pc/mascot/cgi/master_results_2.pl?file=20180201/F022332.dat;pr.eh=322,322p;pr.page=322;pr.per_page=1;pr.show=proteins) | 19 | 35752 | 2 (1) | 2 (1) | 0.09 | Solute carrier family 25 member 33 OS=Homo sapiens GN=SLC25A33 PE=1 SV=1 |
| 340 | [FA49A_HUMAN](http://fitgene-cell-pc/mascot/cgi/master_results_2.pl?file=20180201/F022332.dat;pr.eh=340,340p;pr.page=340;pr.per_page=1;pr.show=proteins) | 17 | 37688 | 2 (1) | 2 (1) | 0.09 | Protein FAM49A OS=Homo sapiens GN=FAM49A PE=2 SV=1 |
| 341 | [MAGF1_HUMAN](http://fitgene-cell-pc/mascot/cgi/master_results_2.pl?file=20180201/F022332.dat;pr.eh=341,341p;pr.page=341;pr.per_page=1;pr.show=proteins) | 17 | 35200 | 4 (1) | 2 (1) | 0.09 | Melanoma-associated antigen F1 OS=Homo sapiens GN=MAGEF1 PE=1 SV=2 |
| 344 | [AK1A1_HUMAN](http://fitgene-cell-pc/mascot/cgi/master_results_2.pl?file=20180201/F022332.dat;pr.eh=344,344p;pr.page=344;pr.per_page=1;pr.show=proteins) | 16 | 36892 | 2 (1) | 2 (1) | 0.09 | Alcohol dehydrogenase [NADP(+)] OS=Homo sapiens GN=AKR1A1 PE=1 SV=3 |
| 160 | [SRSF6_HUMAN](http://fitgene-cell-pc/mascot/cgi/master_results_2.pl?file=20180201/F022332.dat;pr.eh=160,160p;pr.page=160;pr.per_page=1;pr.show=proteins) | 44 | 39677 | 5 (1) | 4 (1) | 0.08 | Serine/arginine-rich splicing factor 6 OS=Homo sapiens GN=SRSF6 PE=1 SV=2 |
| 231 | [ARMX3_HUMAN](http://fitgene-cell-pc/mascot/cgi/master_results_2.pl?file=20180201/F022332.dat;pr.eh=231,231p;pr.page=231;pr.per_page=1;pr.show=proteins) | 29 | 42816 | 2 (1) | 2 (1) | 0.08 | Armadillo repeat-containing X-linked protein 3 OS=Homo sapiens GN=ARMCX3 PE=1 SV=1 |
| 297 | [CCNI_HUMAN](http://fitgene-cell-pc/mascot/cgi/master_results_2.pl?file=20180201/F022332.dat;pr.eh=297,297p;pr.page=297;pr.per_page=1;pr.show=proteins) | 22 | 43157 | 3 (1) | 1 (1) | 0.08 | Cyclin-I OS=Homo sapiens GN=CCNI PE=1 SV=1 |
| 315 | [KERA_HUMAN](http://fitgene-cell-pc/mascot/cgi/master_results_2.pl?file=20180201/F022332.dat;pr.eh=315,315p;pr.page=315;pr.per_page=1;pr.show=proteins) | 20 | 40882 | 11 (1) | 6 (1) | 0.08 | Keratocan OS=Homo sapiens GN=KERA PE=1 SV=1 |
| 206 | [SAHH_HUMAN](http://fitgene-cell-pc/mascot/cgi/master_results_2.pl?file=20180201/F022332.dat;pr.eh=206,206p;pr.page=206;pr.per_page=1;pr.show=proteins) | 35 | 48255 | 7 (1) | 5 (1) | 0.07 | Adenosylhomocysteinase OS=Homo sapiens GN=AHCY PE=1 SV=4 |
| 255 | [TMPS4_HUMAN](http://fitgene-cell-pc/mascot/cgi/master_results_2.pl?file=20180201/F022332.dat;pr.eh=255,255p;pr.page=255;pr.per_page=1;pr.show=proteins) | 27 | 49127 | 3 (1) | 2 (1) | 0.07 | Transmembrane protease serine 4 OS=Homo sapiens GN=TMPRSS4 PE=1 SV=2 |
| 271 | [ASSY_HUMAN](http://fitgene-cell-pc/mascot/cgi/master_results_2.pl?file=20180201/F022332.dat;pr.eh=271,271p;pr.page=271;pr.per_page=1;pr.show=proteins) | 26 | 46786 | 5 (1) | 5 (1) | 0.07 | Argininosuccinate synthase OS=Homo sapiens GN=ASS1 PE=1 SV=2 |
| 303 | [DXO_HUMAN](http://fitgene-cell-pc/mascot/cgi/master_results_2.pl?file=20180201/F022332.dat;pr.eh=303,303p;pr.page=303;pr.per_page=1;pr.show=proteins) | 21 | 45356 | 4 (1) | 3 (1) | 0.07 | Decapping and exoribonuclease protein OS=Homo sapiens GN=DXO PE=2 SV=2 |
| 313 | [MRM3_HUMAN](http://fitgene-cell-pc/mascot/cgi/master_results_2.pl?file=20180201/F022332.dat;pr.eh=313,313p;pr.page=313;pr.per_page=1;pr.show=proteins) | 20 | 47276 | 5 (1) | 5 (1) | 0.07 | rRNA methyltransferase 3, mitochondrial OS=Homo sapiens GN=MRM3 PE=1 SV=2 |
| 343 | [KCD16_HUMAN](http://fitgene-cell-pc/mascot/cgi/master_results_2.pl?file=20180201/F022332.dat;pr.eh=343,343p;pr.page=343;pr.per_page=1;pr.show=proteins) | 16 | 49962 | 3 (1) | 2 (1) | 0.07 | BTB/POZ domain-containing protein KCTD16 OS=Homo sapiens GN=KCTD16 PE=1 SV=1 |
| 351 | [RHBL1_HUMAN](http://fitgene-cell-pc/mascot/cgi/master_results_2.pl?file=20180201/F022332.dat;pr.eh=351,351p;pr.page=351;pr.per_page=1;pr.show=proteins) | 15 | 48796 | 3 (1) | 2 (1) | 0.07 | Rhomboid-related protein 1 OS=Homo sapiens GN=RHBDL1 PE=2 SV=1 |
| 186 | [GLCTK_HUMAN](http://fitgene-cell-pc/mascot/cgi/master_results_2.pl?file=20180201/F022332.dat;pr.eh=186,186p;pr.page=186;pr.per_page=1;pr.show=proteins) | 38 | 55503 | 5 (1) | 3 (1) | 0.06 | Glycerate kinase OS=Homo sapiens GN=GLYCTK PE=1 SV=1 |
| 188 | [FSCN1_HUMAN](http://fitgene-cell-pc/mascot/cgi/master_results_2.pl?file=20180201/F022332.dat;pr.eh=188,188p;pr.page=188;pr.per_page=1;pr.show=proteins) | 38 | 55123 | 3 (1) | 3 (1) | 0.06 | Fascin OS=Homo sapiens GN=FSCN1 PE=1 SV=3 |
| 209 | [DDX6_HUMAN](http://fitgene-cell-pc/mascot/cgi/master_results_2.pl?file=20180201/F022332.dat;pr.eh=209,209p;pr.page=209;pr.per_page=1;pr.show=proteins) | 34 | 54781 | 3 (1) | 3 (1) | 0.06 | Probable ATP-dependent RNA helicase DDX6 OS=Homo sapiens GN=DDX6 PE=1 SV=2 |
| 261 | [ONEC2_HUMAN](http://fitgene-cell-pc/mascot/cgi/master_results_2.pl?file=20180201/F022332.dat;pr.eh=261,261p;pr.page=261;pr.per_page=1;pr.show=proteins) | 26 | 54611 | 2 (1) | 2 (1) | 0.06 | One cut domain family member 2 OS=Homo sapiens GN=ONECUT2 PE=2 SV=2 |
| 304 | [SMAD6_HUMAN](http://fitgene-cell-pc/mascot/cgi/master_results_2.pl?file=20180201/F022332.dat;pr.eh=304,304p;pr.page=304;pr.per_page=1;pr.show=proteins) | 21 | 54319 | 1 (1) | 1 (1) | 0.06 | Mothers against decapentaplegic homolog 6 OS=Homo sapiens GN=SMAD6 PE=1 SV=2 |
| 326 | [GBRD_HUMAN](http://fitgene-cell-pc/mascot/cgi/master_results_2.pl?file=20180201/F022332.dat;pr.eh=326,326p;pr.page=326;pr.per_page=1;pr.show=proteins) | 19 | 50904 | 1 (1) | 1 (1) | 0.06 | Gamma-aminobutyric acid receptor subunit delta OS=Homo sapiens GN=GABRD PE=1 SV=2 |
| 347 | [RGF1B_HUMAN](http://fitgene-cell-pc/mascot/cgi/master_results_2.pl?file=20180201/F022332.dat;pr.eh=347,347p;pr.page=347;pr.per_page=1;pr.show=proteins) | 16 | 55894 | 1 (1) | 1 (1) | 0.06 | Ras-GEF domain-containing family member 1B OS=Homo sapiens GN=RASGEF1B PE=1 SV=2 |
| 349 | [NOXA1_HUMAN](http://fitgene-cell-pc/mascot/cgi/master_results_2.pl?file=20180201/F022332.dat;pr.eh=349,349p;pr.page=349;pr.per_page=1;pr.show=proteins) | 16 | 51586 | 3 (1) | 3 (1) | 0.06 | NADPH oxidase activator 1 OS=Homo sapiens GN=NOXA1 PE=1 SV=1 |
| 357 | [ANGP4_HUMAN](http://fitgene-cell-pc/mascot/cgi/master_results_2.pl?file=20180201/F022332.dat;pr.eh=357,357p;pr.page=357;pr.per_page=1;pr.show=proteins) | 15 | 57326 | 2 (1) | 2 (1) | 0.06 | Angiopoietin-4 OS=Homo sapiens GN=ANGPT4 PE=1 SV=1 |
| 5.5 | [KBTB3_HUMAN](http://fitgene-cell-pc/mascot/cgi/master_results_2.pl?file=20180201/F022332.dat;pr.eh=5,5p;pr.page=5;pr.per_page=1;pr.show=proteins) | 41 | 70775 | 8 (4) | 2 (1) | 0.05 | Kelch repeat and BTB domain-containing protein 3 OS=Homo sapiens GN=KBTBD3 PE=2 SV=2 |
| 241 | [COQ8A_HUMAN](http://fitgene-cell-pc/mascot/cgi/master_results_2.pl?file=20180201/F022332.dat;pr.eh=241,241p;pr.page=241;pr.per_page=1;pr.show=proteins) | 28 | 72418 | 7 (1) | 6 (1) | 0.05 | Atypical kinase COQ8A, mitochondrial OS=Homo sapiens GN=COQ8A PE=1 SV=1 |
| 245 | [ZN695_HUMAN](http://fitgene-cell-pc/mascot/cgi/master_results_2.pl?file=20180201/F022332.dat;pr.eh=245,245p;pr.page=245;pr.per_page=1;pr.show=proteins) | 28 | 62333 | 3 (1) | 3 (1) | 0.05 | Zinc finger protein 695 OS=Homo sapiens GN=ZNF695 PE=1 SV=4 |
| 264 | [FKB10_HUMAN](http://fitgene-cell-pc/mascot/cgi/master_results_2.pl?file=20180201/F022332.dat;pr.eh=264,264p;pr.page=264;pr.per_page=1;pr.show=proteins) | 26 | 64717 | 5 (1) | 2 (1) | 0.05 | Peptidyl-prolyl cis-trans isomerase FKBP10 OS=Homo sapiens GN=FKBP10 PE=1 SV=1 |
| 294 | [GRK5_HUMAN](http://fitgene-cell-pc/mascot/cgi/master_results_2.pl?file=20180201/F022332.dat;pr.eh=294,294p;pr.page=294;pr.per_page=1;pr.show=proteins) | 23 | 68713 | 2 (1) | 2 (1) | 0.05 | G protein-coupled receptor kinase 5 OS=Homo sapiens GN=GRK5 PE=1 SV=1 |
| 310 | [GGA2_HUMAN](http://fitgene-cell-pc/mascot/cgi/master_results_2.pl?file=20180201/F022332.dat;pr.eh=310,310p;pr.page=310;pr.per_page=1;pr.show=proteins) | 20 | 67621 | 3 (1) | 2 (1) | 0.05 | ADP-ribosylation factor-binding protein GGA2 OS=Homo sapiens GN=GGA2 PE=1 SV=3 |
| 328 | [TT39A_HUMAN](http://fitgene-cell-pc/mascot/cgi/master_results_2.pl?file=20180201/F022332.dat;pr.eh=328,328p;pr.page=328;pr.per_page=1;pr.show=proteins) | 18 | 70702 | 4 (1) | 4 (1) | 0.05 | Tetratricopeptide repeat protein 39A OS=Homo sapiens GN=TTC39A PE=2 SV=1 |
| 334 | [NT5D3_HUMAN](http://fitgene-cell-pc/mascot/cgi/master_results_2.pl?file=20180201/F022332.dat;pr.eh=334,334p;pr.page=334;pr.per_page=1;pr.show=proteins) | 18 | 63721 | 3 (1) | 1 (1) | 0.05 | 5'-nucleotidase domain-containing protein 3 OS=Homo sapiens GN=NT5DC3 PE=1 SV=1 |
| 338 | [CSRN2_HUMAN](http://fitgene-cell-pc/mascot/cgi/master_results_2.pl?file=20180201/F022332.dat;pr.eh=338,338p;pr.page=338;pr.per_page=1;pr.show=proteins) | 17 | 60694 | 2 (1) | 1 (1) | 0.05 | Cysteine/serine-rich nuclear protein 2 OS=Homo sapiens GN=CSRNP2 PE=1 SV=1 |
| 348 | [MSL1_HUMAN](http://fitgene-cell-pc/mascot/cgi/master_results_2.pl?file=20180201/F022332.dat;pr.eh=348,348p;pr.page=348;pr.per_page=1;pr.show=proteins) | 16 | 67771 | 3 (1) | 3 (1) | 0.05 | Male-specific lethal 1 homolog OS=Homo sapiens GN=MSL1 PE=1 SV=3 |
| 359 | [TRXR1_HUMAN](http://fitgene-cell-pc/mascot/cgi/master_results_2.pl?file=20180201/F022332.dat;pr.eh=359,359p;pr.page=359;pr.per_page=1;pr.show=proteins) | 13 | 71832 | 5 (1) | 3 (1) | 0.05 | Thioredoxin reductase 1, cytoplasmic OS=Homo sapiens GN=TXNRD1 PE=1 SV=3 |
| 234 | [ENK21_HUMAN](http://fitgene-cell-pc/mascot/cgi/master_results_2.pl?file=20180201/F022332.dat;pr.eh=234,234p;pr.page=234;pr.per_page=1;pr.show=proteins) | 29 | 80154 | 4 (1) | 3 (1) | 0.04 | Endogenous retrovirus group K member 21 Env polyprotein OS=Homo sapiens GN=ERVK-21 PE=1 SV=1 |
| 266 | [TTC14_HUMAN](http://fitgene-cell-pc/mascot/cgi/master_results_2.pl?file=20180201/F022332.dat;pr.eh=266,266p;pr.page=266;pr.per_page=1;pr.show=proteins) | 26 | 88778 | 2 (1) | 1 (1) | 0.04 | Tetratricopeptide repeat protein 14 OS=Homo sapiens GN=TTC14 PE=1 SV=1 |
| 283 | [SRAC1_HUMAN](http://fitgene-cell-pc/mascot/cgi/master_results_2.pl?file=20180201/F022332.dat;pr.eh=283,283p;pr.page=283;pr.per_page=1;pr.show=proteins) | 25 | 74898 | 3 (1) | 2 (1) | 0.04 | Protein SERAC1 OS=Homo sapiens GN=SERAC1 PE=1 SV=1 |
| 284 | [PPCEL_HUMAN](http://fitgene-cell-pc/mascot/cgi/master_results_2.pl?file=20180201/F022332.dat;pr.eh=284,284p;pr.page=284;pr.per_page=1;pr.show=proteins) | 25 | 84843 | 5 (1) | 5 (1) | 0.04 | Prolyl endopeptidase-like OS=Homo sapiens GN=PREPL PE=1 SV=1 |
| 295 | [ZN337_HUMAN](http://fitgene-cell-pc/mascot/cgi/master_results_2.pl?file=20180201/F022332.dat;pr.eh=295,295p;pr.page=295;pr.per_page=1;pr.show=proteins) | 23 | 89157 | 5 (1) | 3 (1) | 0.04 | Zinc finger protein 337 OS=Homo sapiens GN=ZNF337 PE=1 SV=2 |
| 317 | [UBP49_HUMAN](http://fitgene-cell-pc/mascot/cgi/master_results_2.pl?file=20180201/F022332.dat;pr.eh=317,317p;pr.page=317;pr.per_page=1;pr.show=proteins) | 20 | 80687 | 2 (1) | 2 (1) | 0.04 | Ubiquitin carboxyl-terminal hydrolase 49 OS=Homo sapiens GN=USP49 PE=1 SV=1 |
| 342 | [CBPZ_HUMAN](http://fitgene-cell-pc/mascot/cgi/master_results_2.pl?file=20180201/F022332.dat;pr.eh=342,342p;pr.page=342;pr.per_page=1;pr.show=proteins) | 17 | 74577 | 4 (1) | 3 (1) | 0.04 | Carboxypeptidase Z OS=Homo sapiens GN=CPZ PE=1 SV=2 |
| 350 | [ZBED5_HUMAN](http://fitgene-cell-pc/mascot/cgi/master_results_2.pl?file=20180201/F022332.dat;pr.eh=350,350p;pr.page=350;pr.per_page=1;pr.show=proteins) | 16 | 80001 | 9 (1) | 5 (1) | 0.04 | Zinc finger BED domain-containing protein 5 OS=Homo sapiens GN=ZBED5 PE=2 SV=2 |
| 354 | [INT10_HUMAN](http://fitgene-cell-pc/mascot/cgi/master_results_2.pl?file=20180201/F022332.dat;pr.eh=354,354p;pr.page=354;pr.per_page=1;pr.show=proteins) | 15 | 83209 | 3 (1) | 2 (1) | 0.04 | Integrator complex subunit 10 OS=Homo sapiens GN=INTS10 PE=1 SV=2 |
| 258 | [CRNL1_HUMAN](http://fitgene-cell-pc/mascot/cgi/master_results_2.pl?file=20180201/F022332.dat;pr.eh=258,258p;pr.page=258;pr.per_page=1;pr.show=proteins) | 26 | 100902 | 3 (1) | 2 (1) | 0.03 | Crooked neck-like protein 1 OS=Homo sapiens GN=CRNKL1 PE=1 SV=4 |
| 268 | [CHRD_HUMAN](http://fitgene-cell-pc/mascot/cgi/master_results_2.pl?file=20180201/F022332.dat;pr.eh=268,268p;pr.page=268;pr.per_page=1;pr.show=proteins) | 26 | 104703 | 2 (2) | 1 (1) | 0.03 | Chordin OS=Homo sapiens GN=CHRD PE=1 SV=2 |
| 324 | [GANAB_HUMAN](http://fitgene-cell-pc/mascot/cgi/master_results_2.pl?file=20180201/F022332.dat;pr.eh=324,324p;pr.page=324;pr.per_page=1;pr.show=proteins) | 19 | 107263 | 1 (1) | 1 (1) | 0.03 | Neutral alpha-glucosidase AB OS=Homo sapiens GN=GANAB PE=1 SV=3 |
| 332 | [TMC3_HUMAN](http://fitgene-cell-pc/mascot/cgi/master_results_2.pl?file=20180201/F022332.dat;pr.eh=332,332p;pr.page=332;pr.per_page=1;pr.show=proteins) | 18 | 126461 | 3 (1) | 3 (1) | 0.03 | Transmembrane channel-like protein 3 OS=Homo sapiens GN=TMC3 PE=2 SV=3 |
| 158 | [TNIK_HUMAN](http://fitgene-cell-pc/mascot/cgi/master_results_2.pl?file=20180201/F022332.dat;pr.eh=158,158p;pr.page=158;pr.per_page=1;pr.show=proteins) | 44 | 155361 | 12 (1) | 10 (1) | 0.02 | TRAF2 and NCK-interacting protein kinase OS=Homo sapiens GN=TNIK PE=1 SV=1 |
| 285 | [CAMP1_HUMAN](http://fitgene-cell-pc/mascot/cgi/master_results_2.pl?file=20180201/F022332.dat;pr.eh=285,285p;pr.page=285;pr.per_page=1;pr.show=proteins) | 24 | 179230 | 7 (1) | 6 (1) | 0.02 | Calmodulin-regulated spectrin-associated protein 1 OS=Homo sapiens GN=CAMSAP1 PE=1 SV=2 |
| 286 | [TDR12_HUMAN](http://fitgene-cell-pc/mascot/cgi/master_results_2.pl?file=20180201/F022332.dat;pr.eh=286,286p;pr.page=286;pr.per_page=1;pr.show=proteins) | 24 | 134717 | 8 (1) | 7 (1) | 0.02 | Putative ATP-dependent RNA helicase TDRD12 OS=Homo sapiens GN=TDRD12 PE=2 SV=2 |
| 308 | [CRBG1_HUMAN](http://fitgene-cell-pc/mascot/cgi/master_results_2.pl?file=20180201/F022332.dat;pr.eh=308,308p;pr.page=308;pr.per_page=1;pr.show=proteins) | 21 | 190097 | 7 (1) | 6 (1) | 0.02 | Beta/gamma crystallin domain-containing protein 1 OS=Homo sapiens GN=CRYBG1 PE=1 SV=3 |
| 335 | [FLIP1_HUMAN](http://fitgene-cell-pc/mascot/cgi/master_results_2.pl?file=20180201/F022332.dat;pr.eh=335,335p;pr.page=335;pr.per_page=1;pr.show=proteins) | 18 | 138537 | 12 (1) | 11 (1) | 0.02 | Filamin-A-interacting protein 1 OS=Homo sapiens GN=FILIP1 PE=1 SV=1 |
| 345 | [M3K4_HUMAN](http://fitgene-cell-pc/mascot/cgi/master_results_2.pl?file=20180201/F022332.dat;pr.eh=345,345p;pr.page=345;pr.per_page=1;pr.show=proteins) | 16 | 183280 | 10 (1) | 8 (1) | 0.02 | Mitogen-activated protein kinase kinase kinase 4 OS=Homo sapiens GN=MAP3K4 PE=1 SV=2 |
| 346 | [CO4A3_HUMAN](http://fitgene-cell-pc/mascot/cgi/master_results_2.pl?file=20180201/F022332.dat;pr.eh=346,346p;pr.page=346;pr.per_page=1;pr.show=proteins) | 16 | 163080 | 9 (1) | 9 (1) | 0.02 | Collagen alpha-3(IV) chain OS=Homo sapiens GN=COL4A3 PE=1 SV=3 |
| 180 | [EMAL6_HUMAN](http://fitgene-cell-pc/mascot/cgi/master_results_2.pl?file=20180201/F022332.dat;pr.eh=180,180p;pr.page=180;pr.per_page=1;pr.show=proteins) | 38 | 220270 | 9 (1) | 7 (1) | 0.01 | Echinoderm microtubule-associated protein-like 6 OS=Homo sapiens GN=EML6 PE=2 SV=2 |
| 262 | [DYHC2_HUMAN](http://fitgene-cell-pc/mascot/cgi/master_results_2.pl?file=20180201/F022332.dat;pr.eh=262,262p;pr.page=262;pr.per_page=1;pr.show=proteins) | 26 | 495790 | 21 (1) | 16 (1) | 0.01 | Cytoplasmic dynein 2 heavy chain 1 OS=Homo sapiens GN=DYNC2H1 PE=1 SV=4 |
| 275 | [ASPM_HUMAN](http://fitgene-cell-pc/mascot/cgi/master_results_2.pl?file=20180201/F022332.dat;pr.eh=275,275p;pr.page=275;pr.per_page=1;pr.show=proteins) | 25 | 413189 | 20 (1) | 19 (1) | 0.01 | Abnormal spindle-like microcephaly-associated protein OS=Homo sapiens GN=ASPM PE=1 SV=2 |
| 279 | [TLN1_HUMAN](http://fitgene-cell-pc/mascot/cgi/master_results_2.pl?file=20180201/F022332.dat;pr.eh=279,279p;pr.page=279;pr.per_page=1;pr.show=proteins) | 25 | 271766 | 15 (1) | 14 (1) | 0.01 | Talin-1 OS=Homo sapiens GN=TLN1 PE=1 SV=3 |
| 318 | [ATRX_HUMAN](http://fitgene-cell-pc/mascot/cgi/master_results_2.pl?file=20180201/F022332.dat;pr.eh=318,318p;pr.page=318;pr.per_page=1;pr.show=proteins) | 20 | 284863 | 17 (1) | 11 (1) | 0.01 | Transcriptional regulator ATRX OS=Homo sapiens GN=ATRX PE=1 SV=5 |
| 339 | [IGS10_HUMAN](http://fitgene-cell-pc/mascot/cgi/master_results_2.pl?file=20180201/F022332.dat;pr.eh=339,339p;pr.page=339;pr.per_page=1;pr.show=proteins) | 17 | 292936 | 20 (1) | 6 (1) | 0.01 | Immunoglobulin superfamily member 10 OS=Homo sapiens GN=IGSF10 PE=1 SV=1 |
| 356 | [SPTCS_HUMAN](http://fitgene-cell-pc/mascot/cgi/master_results_2.pl?file=20180201/F022332.dat;pr.eh=356,356p;pr.page=356;pr.per_page=1;pr.show=proteins) | 15 | 282681 | 8 (1) | 5 (1) | 0.01 | Spatacsin OS=Homo sapiens GN=SPG11 PE=1 SV=3 |
| 358 | [ESPL1_HUMAN](http://fitgene-cell-pc/mascot/cgi/master_results_2.pl?file=20180201/F022332.dat;pr.eh=358,358p;pr.page=358;pr.per_page=1;pr.show=proteins) | 14 | 236564 | 7 (1) | 6 (1) | 0.01 | Separin OS=Homo sapiens GN=ESPL1 PE=1 SV=3 |
| 309 | [MUC19_HUMAN](http://fitgene-cell-pc/mascot/cgi/master_results_2.pl?file=20180201/F022332.dat;pr.eh=309,309p;pr.page=309;pr.per_page=1;pr.show=proteins) | 20 | 812409 | 12 (1) | 8 (1) | 0.01 | Mucin-19 OS=Homo sapiens GN=MUC19 PE=1 SV=3 |
