# Supplementary material for: LncRNA H19 Overexpression Activates Wnt Signaling to Maintain the Hair Follicle Regeneration Potential of Dermal Papilla Cells
Source: Front Genet. 2020 Aug 4;11:694. doi: 10.3389/fgene.2020.00694 (PMC7417632; doi:10.3389/fgene.2020.00694)
Supplement: FILE S1 — The detailed procedure of H19 overexpression lentivirus construction. [file Data_Sheet_1.doc]

### Construction of the recombinant LV-H19 lentivirus vector

The H19 overexpression vector-GV367 containing the full-length H19 (NR_002196) sequence were constructed by Shanghai GeneChem Co. , Ltd (18952-1, China) , following the Lentivector User Manual. Top 10 competent cells were transformed and positive clones were identified by PCR. After the PCR products were sequenced, positive clones were chosen to propagate. The plasmid was extracted using Granule Extraction Kit according to the manufacturer’s instructions (Tiangen, DP117, China ). To package the lentiviruses, 293T packaging cells were plated in a 10 cm plate. At 70% confluency, the cells were co-transfected with prepared DNA solutions (GV vector plasmid 20 μg, Helper 1.0 vector plasmid 15 μg, helper 2.0 vector plasmid 10 μg) using the transfection kit from GeneChem Co. , Ltd. The viral supernatants were harvested after 48h and filtered using a 0.45 μM filter. The titer of the lentivirus was determined by real-time PCR.
